# Supplementary material for: A Systematic Review on Metabolomics Analysis in Hearing Impairment: Is It a Possible Tool in Understanding Auditory Pathologies?
Source: Int J Mol Sci. 2023 Oct 14;24(20):15188. doi: 10.3390/ijms242015188 (PMC10607298; doi:10.3390/ijms242015188)
Supplement: Supplementary file 1 [file ijms-24-15188-s001.zip › ijms-2589827-supplementary.pdf]

Supplementary Table S1: The technologies used in metabolomics studies, their full names, and their advantages and disadvantages for both targeted and untargeted metabolomics

Technology	Full Name	Advantages for Targeted Metabolomics	Disadvantages for Targeted Metabolomics	Advantages for Untargeted Metabolomics	Disadvantages for Untargeted Metabolomics
**LC-MS**	Liquid Chromatography-Mass Spectrometry	High sensitivity, broad metabolite coverage	Limited to pre-defined metabolites	Comprehensive profiling, diverse metabolites	Data complexity, challenges in annotation
**LC-MS/MS**	Liquid Chromatography-Tandem Mass Spectrometry	Improved selectivity and sensitivity	Targeted approach, potential for missing metabolites	Broad metabolite coverage, structural info	Complexity, need for extensive databases
**GC-MS**	Gas Chromatography-Mass Spectrometry	High resolution, stability of GC column	Limited to volatile and thermally stable compounds	Excellent quantification, reproducibility	Limited to volatile compounds, derivatization needed
**HILIC-UHPLC-QTOF–MS**	Hydrophilic Interaction Liquid Chromatography- Ultra high pressure – quadrupole and time of flight MS	Separation of polar metabolites	Reduced coverage of non-polar compounds	Comprehensive separation of polar compounds	Limited separation of non-polar metabolites
**LC-ESI-MS**	Liquid Chromatography-Electrospray Ionization-MS	High sensitivity, ionization of broad metabolite range	Variable ionization efficiency, matrix effects	Comprehensive profiling, wide metabolite range	Ion suppression, interference from complex matrices
**HPLC-MS/MS**	High Performance Liquid Chromatography-MS/MS	High specificity and sensitivity	Limited to pre-defined metabolites, fragmentation limits	Targeted analysis with high accuracy and selectivity	Narrow scope, challenges in comprehensive profiling
**UPLC-MS/MS**	Ultra Performance Liquid Chromatography-MS/MS	Improved separation, high throughput	Targeted approach, potential for missing metabolites	Broad metabolite coverage, enhanced sensitivity	Equipment cost, complexity of analysis
**GC-TOF/MS**	Gas Chromatography-Time of Flight Mass Spectrometry	High resolution, broad coverage	Limited to volatile compounds	Comprehensive profiling, accurate mass measurement	Sample derivatization, limited to volatile compounds
**GC-HRMS**	Gas Chromatography-High Resolution Mass Spectrometry	High resolution, accurate mass measurement	Limited to volatile compounds	Broad metabolite coverage, structural info	Limited to volatile compounds, data complexity

**UHPLC-QTOF MS**	Ultra High-Performance Liquid Chromatography-QTOF MS	High resolution, accurate mass measurement	Limited to specific chromatographic conditions	Comprehensive profiling, accurate mass measurement	Variable performance depending on instrument settings
**LC-HRMS**	Liquid Chromatography-High Resolution Mass Spectrometry	High resolution, accurate mass measurement	Variable performance depending on instrument settings	Broad metabolite coverage, accurate mass measurement	Sample complexity, ion suppression
**NMR**	Nuclear Magnetic Resonance	Non-destructive, quantitative without standards	Lower sensitivity, limited dynamic range	Comprehensive profiling, non-destructive	Limited sensitivity, spectral overlap
